# A Fable

**Published:** 1887-10

**Authors:** 


A FABLE.—A Recent Graduate, who had but lately ceased to manipulate a plow, was basking in abundant leisure, when he was accosted by a Lacerated Uterus.

"Are you a Doctor?" asked the Uterus.

"Yes," replied the Recent Graduate, "let me sew you up."

"Hands off!" exclaimed the Lacerated Uterus, holding up her fallopian tubes in horror. "I have been sewed up too much already, and what I come here for is to know why you doctors can't let me alone. Once I was young and handsome (here the Lacerated Uterus sighed so loudly that the Recent Graduate murmured 'Physometra'), but a long course of Local Treatment, injections, swabbings, applications and operations have left me in this disfigured condition. Why are all the ills of humanity heaped upon my neck?" continued the Uterus, wiping her lips with the fringed extremity of her left Fallopian tube. "Why am I responsible for everything from Consumption to Corns?"

At this moment a Rectum came strolling along with his hands in his pockets, just in time to hear the last remark of the Lacerated Uterus.

"Rats, Sister!" exclaimed the Rectum.

"Prats, you mean," said the Recent Graduate, at which the Rectum winked, but he continued.

"Rats, Sister! it is I with my little Pockets that have to bear everything. My Papillæ are cut off for Paralysis, and my Pockets are cut out for Boils, and my Sphincter is stretched for Headaches, and I am maltreated in every way for the ills of other Organs." Here the Rectum sighed in an audible manner.

"Pockets! Papillæ!" exclaimed the Recent Graduate in a frenzied tone. "Great Heavens! Let me cut them out."

"Not much," said the Rectum, as he rubbed one of his Piles in a soothing way. "I have seen too much of it already. Only yesterday my brother arose from his downy bed after such an operation, very much disfigured, but still in the Ring."

"Brother," said the Lacerated Uterus, "possibly your words are true, and I am going to have a Protracted Rest. But I must be going; will your Hæmorrhoidal Highness accompany me?"

"Certes," said the Rectum, with a smile upon his wrinkled countenance, and together they went out, leaving the Recent Graduate searching his pocket with an air of anxiety for a nickel wherewith to purchase Beer.

"I would I had some Gold, besides Aurum 30x," sighed he, as he failed to find the Elusive Coin. Then, falling into an empty chair, the Recent Graduate assumed an attitude of Acute Despair. —*Med. Vis.*

